# Effectiveness and costs of interventions to reduce the within-farm *Toxoplasma gondii* seroprevalence on pig farms in the Netherlands

**DOI:** 10.1186/s40813-021-00223-0

**Published:** 2021-07-26

**Authors:** Dorien M. Eppink, Henk J. Wisselink, Inge M. Krijger, Joke W.B. van der Giessen, Manon Swanenburg, Coen P.A. van Wagenberg, Marcel A.P.M. van Asseldonk, Martijn Bouwknegt

**Affiliations:** 1 Van Hall Larenstein, Velp, The Netherlands; 2 Wageningen Bioveterinary Research, Lelystad, The Netherlands; 3 Kennis- en Adviescentrum Dierplagen, Wageningen, The Netherlands; 4 National Institute for Public Health and the Environment, Bilthoven, The Netherlands; 5 Wageningen Economic Research, Wageningen, The Netherlands; 6 Vion Food Group, Boxtel, The Netherlands

**Keywords:** *Toxoplasma gondii*, Pigs, Interventions, Risk factors, Seroprevalence, Cats, Rodent control, Audits

## Abstract

**Background:**

The parasite *Toxoplasma gondii (T. gondii)* is recognized as one of the major foodborne pathogens with a high human disease burden. To control *T. gondii* infections in pigs, European Food Safety Agency (EFSA) advises serological testing of pigs and audits of pig farms to identify risk factors for *T. gondii* infection. In line with this approach, the aim of the current study was to assess the effectiveness and costs of intervention measures implemented to reduce the *T. gondii* seroprevalence on finishing pig farms in the Netherlands. A crossover clinical trial was conducted at five case farms were their own control and the cross-over moment was the implementation of interventions to reduce risk factors. Each of the case farms had a farm-specific intervention strategy with one principal intervention measure (neutering of cats, professional rodent control or covering food storage).

**Results:**

All finishing pig farms (*n* = 5) showed a reduction in *T. gondii* seroprevalence within one year of implementing the intervention strategy. Cat neutering (*n* = 3) and feed coverage (*n* = 1) showed statistically significant reductions in seroprevalence. Rodent control (*n* = 1) did not show a statistically significant reduction. The estimated reduction in seroprevalence in response to the neutering of cats and feed coverage were 67 and 96 %, respectively.

**Conclusions:**

Our work demonstrates that it is possible to reduce the within-farm *T. gondii* seroprevalence within one year after interventions were implemented to reduce *T. gondii* risk factors. This information is essential and encouraging for policy makers, food business operators, and farmers to implement in their risk assessment and to apply to food safety control systems.

**Supplementary Information:**

The online version contains supplementary material available at 10.1186/s40813-021-00223-0.

## Background

*Toxoplasma gondii* (*T. gondii*) is an ubiquitous protozoan parasite capable of infecting virtually all warm-blooded vertebrates throughout the world [1, 2]. It is an obligate intracellular organism belonging to the coccidian family. The parasite has a complex life cycle whereby *Felidae* (cats) function as the definitive host. Cats can shed millions of oocysts after a primary infection into the environment via their feces during a period of a few weeks [1]. Intermediate hosts can become infected after ingesting soil, water or feed, contaminated with oocysts. After infection, oocysts transform into tachyzoites in the host. This life stage spreads throughout the host and eventually localizes primarily in neural and muscle tissue where it develops into tissue cyst bradyzoites. Intermediate hosts include (wild) animals such as birds and rodents, and also farm animals such as pigs. Humans can get infected via oocysts present on unwashed hands after gardening or infrequently cleaning the cat litter when oocysts are present, consuming raw or undercooked meat containing tissue cysts, food or water contaminated with oocysts, and to a lesser extend also by blood or organ transfusion or transplacental from mother to fetus. A *T. gondii* infection can cause severe symptoms in humans with immature or impaired immune systems such as fetuses or immunocompromised patients [1]. In non-immunocompromised humans, a postnatal infection can cause ocular disease [3].

*T. gondii* is recognized as an important foodborne zoonosis, with a high global public health impact estimated at 1.20 million disabilities adjusted life years (DALYs) annually for congenital toxoplasmosis [4]. In 2015, *T. gondii* ranked fourth out of 24 foodborne parasites globally [5]. In Europe, *T. gondii* ranked second out of 23 foodborne parasites [6]. Among the different food sources of animal origin, pork is seen as an important source for *T. gondii* infection [7]. In the Netherlands, 12 % of human infections were attributed to consumption of pork meat [8]. Viable *T. gondii* parasites have been isolated from tissues and meat of pigs, both naturally and experimentally infected with *T. gondii* [1]. *T. gondii* infections in pigs are commonly asymptomatic, however, clinical signs can occur during the acute phase of infection [1]. The level of *T. gondii* infections in pig herds depends on the farming system, with outdoor access leading to a higher seroprevalence compared to only being held indoors [9–11]. The risk for *T. gondii* infections in pigs is associated with the presence of cats, occurrence of rodents, accessibility of cats and rodents to pig feed and water and the degree of cleaning and disinfection on the farm [12–18].

Given the high human disease burden associated with *T. gondii*, preventive interventions are needed, but so far no control in meat producing animals is being carried out since tissue cysts are not visible during meat inspection. To control *T. gondii* infections in the pork supply chain, the European Food Safety Authority (EFSA) has developed a set of epidemiological indicators combined with a package of recommended measures [9]. Proposed measures are serological testing of pigs for *T. gondii* infections at the farms or slaughterhouse, and on farm audits for risk factors associated with a *T. gondii* infection. In line with the EFSA recommendations a large-scale private serological monitoring program was started in a pig slaughter company in the Netherlands in 2012. From every delivery of pigs to a slaughterhouse of the company blood samples were taken. The seroprevalence in these pig herds over farming systems, years, regions and seasons are described in Swanenburg et al. [11]. Using these data, farms with a high seroprevalence were identified. Intervention measures were needed to control *T. gondii* infections in these herds. However, information on the effectiveness and costs of intervention measures to control *T. gondii* infections in pigs on farms is scarce. This information is essential for policy makers, food business operators and farmers to implement in their risk assessment and to apply into food safety control systems. The aim of the current study was to assess the effectiveness and costs of intervention measures to reduce the *T. gondii* seroprevalence in the finishing pigs on farms in the Netherlands.

## Results

### Farm characteristics

Nine farmers were approached for this study, each farm with an estimated within farm seroprevalence > 10 % and a minimum of six deliveries in the previous running year, i.e. the selection period. Five farmers were willing to participate in the study. These five farms had 917 finishing pigs on average, with a range from 100 to 3,333 (Table 1). Two farms were managed conventionally, and three organically. Starting dates of the interventions ranged from 25 to 2017 to 1 March 2018. Taken together the results of all pigs from the five farms, 1,280 blood samples were analyzed during the two-year study period, of which 258 (20.2 %) tested positive for the presence of *T. gondii* antibodies.

**Table 1:** Farm characteristics and study data of the five pig farms participating in the intervention study

Farm #	Farm type	Farm size (# finishing pigs)	Farm system	Start date of intervention strategy	# Blood samples before intervention (positive; total; %)	# Blood samples after intervention (positive; total; %)
1	Finisher	3333	Conventional	25 Aug. 2017	138 ; 278 ; 50	65 ; 320 ; 20
2	Breeder-Finisher	600	Organic	15 Nov. 2017	16 ; 150 ; 11	1 ; 126 ; 1
3	Finisher	200	Organic	28 Dec. 2017	6 ; 83 ; 7^a^	4 ; 96 ; 4
4	Finisher	100	Organic	1 Dec. 2017	6 ; 48 ; 13	2 ; 24 ; 8
5	Finisher	350	Conventional	1 Mar. 2018	12 ; 71 ; 17	6 ; 47 ; 13

^a^Due to the fact that the date of selection and the start date of the actual intervention strategy differed the estimated with-in farm seroprevalence turned out to be < 10 % for one farm in the year before the start of the implementation of the intervention strategy

### Interventions implemented on each farm

The intervention strategy differed per farm and was selected by the project team after an initial visit. A specific, principal intervention measure was selected for each farm. On Farm 1, 2 and 3 stray cats were present at the farm during the initial farm visit (Table 2). Therefore, at these farms the chosen principal intervention measure was neutering of all captured cats. At Farm 1 neutering took place on 25 August 2017: eight cats were neutered and two kittens were removed from the premises. The cross over date on Farm 2 was 15 November 2017: seven cats were neutered. On Farm 3 seven cats were neutered at 28 December 2017). Farm 4 showed the lowest score in the initial visit with the Hazard Analysis Critical Control Points (HACCP) based questionnaire on the risk factor presence of rodents (Table 2; 30 %). Traces of rodents were observed in and outside the stables and professional rodent control was not in place (Figure S2). Therefore, implementation of professional rodent control was selected as the principal intervention measure (starting 1 December 2017: 24 visits per year and two periods of one week camera surveillance to provide the farmer insight into the presence of rodents). On Farm 5, the principal intervention measure was the implementation of a covered storage for the whey that was put in place on 1 March 2018. Although there were stray cats present (Table 2), it was decided, for practical reasons, to select feed coverage as the farm’s principal intervention measure, because the farmer had built the new storage. The covered storage was a completely sealed off plastic barrel placed in the week of the initial farm visit (Figure S3). During the intervention period some farms also implemented other intervention measures in addition to the principal intervention measure, e.g. the placement of a new door and paving a trench silo on Farm 1 (Tables 2 and 3, Figure S1).

**Table 2:** Results of the questionnaire (percentage of total points) before and after implementation of the intervention strategy

Topic	Farm 1 (first visit; final visit)	Farm 2 (first visit; final visit)	Farm 3 (first visit; final visit)	Farm 4 (first visit; final visit)	Farm 5 (first visit; final visit)
General farm biosecurity	28 ; 28	22 ; 28	33 ; 33	17 ; 17	28 ; 28
Supply of pigs	33 ; 50	67 ; 67	50 ; 50	33 ; 33	83 ; 83
Outdoor access	100 ; 100	67 ; 67	67 ; 67	67 ; 67	100 ; 100
Presence of cats	0 ; 40	0 ; 40	0 ; 40	80 ; 80	0 ; 0
Feed supply	58 ; 67	83 ; 83	83 ; 83	75 ; 75	67 ; 75
Water supply	50 ; 50	75 ; 75	75 ; 75	75 ; 75	75 ; 75
Pest control and prevention	60 ; 70	40 ; 45	35 ; 70	30 ; 35	45 ; 45

**Table 3:** Costs of interventions implemented on the five pig farms to control *T. gondii* infections

Farm #	Intervention measure^a^		One-off expenses	Labor (hours)	Total one-off expenses^b^	Monthly expenses	Estimated annual costs
1	PIM	Sterilizing eight cats	€115		€115		€25
	OM	Improving rodent control by a professional company visiting the farm every six weeks (including inside the stable)				€200	€2400
	OM	Placing a new door in the stable to prevent mice and rats from entering	€1000		€1000		€131
	OM	Paving trench silo and tidying up between pig stables	€2000	80	€4265		237
		Total	€3115	80	€5380	€200	€2792
2	PIM	Sterilizing seven cats	€420		€420		€91
	OM	Placing a rattrap	€36		€36		€5
	OM	Implementing fly control by using predatory flies and wasps (only during the summer every 5 weeks, in total 7 times)				€310	€3714
	OM	Placing boot cleaners and using new boots	€126		€126		€16
		Total	€582		€582	€310	€3827
3	PIM	Sterilizing seven cats	€505	11	€801		€178
	OM	Improving rodent control by placing poison boxes inside and traps outside every four to six weeks				€101	€1212
		Total	€505	11	€816	€101	€1390
4	PIM	Improving rodent control by a professional company visiting the farm every two to three weeks (including camera surveillance)				€240	€2880
	OM	Placing a lid on feeding cart^c^					
	OM	Cleaning pig stables^c^					
		Total				€240	€2880
5	PIM	Implementing a new storage for whey feed	€4500		€4500		€362
		Total	€4500		€4500		€362

All costs mentioned are excl. VAT

^a^*PIM* principal intervention measure, *OM* other intervention measure

^b^Labor is valued at €28.31 per hour [19]

^c^No data on expenses received from the farmer

### Efficacy of principal intervention measures

**Fig. 1:**
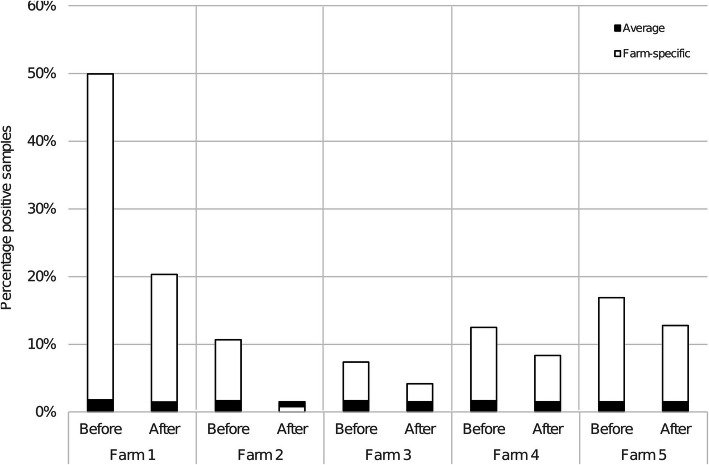
Overview per farm of percentage positive serum samples for *T. gondii* antibodies in the finishing pigs. ‘Before’ indicates the percentage positive in the 12 months before the start of the intervention, ‘After’ indicates the percentage positive in the 12 months after the start of the intervention. The black bars (‘average’) indicate the average percentage *T. gondii* positive serum samples for all slaughter pigs in the Netherlands in the respective ‘before’ and ‘after’ period for each farm (extended results from [11])

Figure 1 shows the development of the percentage of positive serum samples for *T. gondii* antibodies in the finishing pigs delivered by each of the five farms to the slaughter house. All farms showed a reduction in the percentage positive serum samples one year within the start of the implementation of the intervention strategy. In comparison the average percentage of positive serum samples for all pigs delivered to the slaughterhouse during the two-year study period stayed approximately the same (1.6 %).

For two of the three principal intervention measures, a statistically significant association with *T. gondii* seroprevalence was found (*p *= 0.05; Table 4). This resulted in an estimated reduction in seroprevalence of 67 % for cat neutering and 96 % for feed coverage.

**Table 4:** Seroprevalence results per principal intervention measure for the five pig farms during the intervention period

Principal intervention measure	Farm #	Odds Ratio (95% CI)	Reduction in seroprevalence (%)
Cat neutering	1, 2, 3	0.33 (0.22 – 0.45)	67 % (55% – 78%)
Professional rodent control	4	3.00 (0.13 – 14.60)	n.a.^a^
Feed coverage	5	0.04 (-∞ – 0.23)	96 % (77% – 100%)

^a^The estimated reduction in seroprevalence was only calculated for the intervention measure where the OR 95% confidence interval did not include 1

 Results of the HACCP based questionnaire, which was used at quarterly evaluation moments, showed in general a good compliance with the chosen intervention strategy by the participating farmers. All farms had a higher score at the end of the intervention period than at the initial farm visit for the risk factor related to the selected principal intervention measure (Table 2). For example, on Farm 1 the intervention strategy was neutering of cats with the related topic of the questionnaire ‘Presence of cats’. At the start of the study period, the farm scored zero out of five points on this topic (= 0 %) and at the end the farm scored two out of five points (= 40 %).

### Expenses and annual costs of interventions

Total one-off costs of interventions varied between the farms from €600 to €5,300 per farm, of which the largest part consisted of expenditures on equipment or services and a lesser part on own labor (Table 3). Neutering of cats only required one-off expenses varying from €115 to €600 per farm, depending on the number and sex of the cats. Monthly expenses of improved rodent and pest control varied from €100 to €300 per farm. The investment in a new whey storage amounted €4,500. The total estimated annual costs for *T. gondii* risk factors reduction varied from around €350 to €4,000. Monthly recurring expenses for controlling pests such as rodents and flies made up between 85 and 100 % of the annual costs.

## Discussion

This study aimed to examine the effectiveness and costs of intervention measures on pig farms to reduce the *T. gondii* seroprevalence in finishing pigs. Each of the five farms followed in this study had a farm-specific intervention strategy with one principal intervention measure. All farms showed a reduction in percentage of positively tested samples for *T. gondii* antibodies one year after implementation of the intervention strategy. The interventions aimed at reducing cats and improved feed coverage showed statistically significant reductions in seroprevalence. The intervention aimed at rodent control did not show a statistically significant reduction. The estimated reduction in seroprevalence of the neutering of cats and feed coverage were 67 and 96 %, respectively. All together, these results show that it is possible to reduce the within-farm *T. gondii* seroprevalence within one year after implementing interventions to reduce specific *T. gondii* risk factors.

On three farms (1, 2 and 3) neutering of cats was decided to be the principal intervention measure because of the presence of cats at these farms. Since cats are the definitive host of *T. gondii* and they spread *T. gondii* oocysts in the environment, specific interventions to reduce the presence of cats on pig farms belong to the primary intervention methods of interest. A significant reduction in seroprevalence on the three farms was observed (*p* = 0.05) and it was concluded that neutering of cats resulted in a reduced exposure of pigs to *T. gondii* within one year. These results are in agreement with earlier results from Mateus Pinilla et al. [20], who showed that administration of an oral *T. gondii* live vaccine to cats on pig farms also leads to a decrease in *T. gondii* seroprevalence. Although vaccination of cats seems to be as successful as neutering of cats to decrease the *T. gondii* seroprevalence on pig farms, vaccination of cats is currently not feasible due to the non-availability of such a vaccine. Moreover, although promising results were obtained with an oral live vaccine [20], commercial production of this vaccine was discontinued because the vaccine needs to be kept frozen, has a short shelf life, high costs, and the lack of interest by cat owners for this vaccine [21].

Neutering cats was used in this study to reduce the presence of young cats, because they pose the highest risk of spreading oocysts in the environment. Most cats are infected with *T. gondii* as juveniles [19], even suckling kittens were found to be shedding oocysts [22]. Cats only spread *T. gondii* in their feces for 1–3 weeks following the first episode of infection and they become immune to re-shedding of oocysts [21]. Therefore, we decided on neutering to reduce the birth of kittens on the farm as an intervention. This is also socially more acceptable for cat owners than culling of the cats present on the farm. A drawback of this approach can be that farmers need to be attentive to the number of cats present on the farm and which cats are neutered, because stray cats can also have kittens in the direct surroundings of the farm.

After neutering of the cats at the farms, within one year a significant reduction in *T. gondii* seroprevalence in slaughter pigs was observed. This reduction could be caused by the neutering, because it would take about 45 weeks, roughly calculated, to observe such a reduction. The pregnancy period of a cat is typically 65 days (9 weeks), the average age of kittens when they start with shedding oocysts is most probably within 10 weeks of age and the fattening period of the slaughter pigs is 26 weeks. However, the ability of oocysts to survive for a long time in the environment can increase the time before the reduction in seroprevalence due to the neutering of cats can be observed.

On one farm (Farm 4, organic) professional rodent control was applied as the principal intervention measure. However, we did not find a significant contribution of this intervention measure to the reduction of *T. gondii* seroprevalence on this farm. This is not in line with expectations, because the presence of rodents is seen as a risk factor for *T. gondii* infections in pig herds, especially on organic farms. Mice and rats can be infected with *T. gondii* or carry *T. gondii* oocysts with them and spread this in the environment of the pigs [23]. Furthermore, results from a Dutch study on application of rodent control on pig farms showed that rodent control was successful in reducing the *T. gondii* seroprevalence in pigs [24]. That we did not find an impact of rodent control in our study could possibly be because rodents were not the main cause of exposure to *T. gondii* on this farm or the intervention period of one year is too short to observe an effect (in contrast to the cat neutering intervention measure, as discussed above). Alternatively, the professional rodent control in itself might have been insufficient or because effective rodent control might requires additional efforts by the farmer and farm personnel. Regarding the latter, the periodically completed HACCP-based questionnaire showed no improvement of general farm biosecurity by the farmer during the intervention year and that the unfavorable circumstances inside and outside the stables facilitating access of rodents to stables remained unchanged. This could suggest that application of professional rodent control in itself might not always be enough to reduce *T. gondii* seroprevalence in pigs. Previous research from Krijger et al. [25] also found that rodent control was more efficient and effective when using integrated pest management. In addition, rodent control is also important to prevent other diseases such as *Trichinella* infections in pigs and therefore it should always be implemented.

Coverage of feed is also determined as a preventive factor for *T. gondii* infections in pig herds [18]. In the current study, the efficacy of feed coverage was estimated at 96 % (76–100 %) reduction in seroprevalence based on a single farm. Although the percentage in seroprevalence reduction on this farm (Farm 5) was 4 % (from 17 to 13 %), the six positive samples after the intervention had started were collected from a single delivery 13 days after the feed was covered. Therefore, these positive samples received less weight in the statistical analyses, resulting in the statistically significant reduction. The farm changed the storage for dairy cow whey used as feed for pigs from an open tank to a new and completely closed tank. Recently Dubey et al. [26] concluded in a review that there is no evidence for the excretion of viable *T. gondii* in cow’s milk. Thus, the whey was probably contaminated with *T. gondii* by rodents or cats, rather than through the cow’s milk. Therefore, preventing rodents or cats to access feed storages can be a valuable intervention measure to control *T. gondii* infections in pigs.

In this study we used the *T. gondii* seroprevalence in slaughter pigs of 26 weeks of age to assess the effectiveness of the principal intervention measures. However, *T. gondii* seroprevalence can vary over seasons and over years [11]. Therefore, we compared the results of the five farms with the development in *T. gondii* seroprevalence of slaughter pigs of other farms in the Netherlands. This showed that the observed decrease in seroprevalence was specific to these farms and not influenced by seasonal and yearly variations.

During visits at the farms, pig farmers were neither discouraged nor prohibited to implement other intervention measures which could reduce *T. gondii* seroprevalence. Any other intervention measures that were implemented were recorded in the HACCP-based questionnaire. However, the statistical power provided by these observations was too low to properly distinguish the effects of the different intervention measures. The observed reduction in seroprevalence on a farm was fully attributed to the principal intervention measure implemented on that farm. This might have resulted in an overestimation of the effect of the principal intervention measures.

The efficacy of cat neutering was estimated at 67 % reduction in seroprevalence and associated average costs were around €100 per year per farm. However, because other interventions were also implemented on each farm simultaneously, the total annual average costs amounted to €2,670 per year per farm. Van Asseldonk et al. [27] estimated that interventions with 67 % efficacy are cost-effective when the annual costs are less than around €4,000, at a value for a DALY of €50,000. For feed coverage, efficacy was estimated at 96 % reduction in seroprevalence and associated costs at €360 per year on Farm 5. This was the only intervention implemented on this farm. Interventions with 96 % efficacy were estimated to be cost-effective when annual costs are less than around €5,800. This suggests that implementation of cat neutering and feed coverage to control *T. gondii* infections in finishing pigs on the farms in our study was cost-effective at macro level. Although it is of interest at macro level, this could not be the case for individual farmers. A *T. gondii* infection does not impact performance nor does it lead to carcass condemnation. This means that a pig farmer does not have a direct economic benefit from implementing interventions to control *T. gondii* infection in pigs. However, livestock producers also base farm decisions on values, motivations, social influences and behavioral factors [28]. Van Wagenberg et al. [29] concluded that many Dutch pig producers had knowledge about risk sources for and consequences of *T. gondii* infections in pigs, but that the public health impact and risks of *T. gondii* infections in pigs were not yet common knowledge among all Dutch pig producers. Reducing such behavioral barriers could induce pig producers to implement interventions to control *T. gondii* infections in pigs.

## Conclusions

This study showed that it is possible to reduce the *T. gondii* within-farm seroprevalence on pig farms by auditing for the presence of specific *T. gondii* risk factors followed by intervention to reduce these risk factors.

## Materials and methods

### Study design and selection of farms

The presence of antibodies against *T. gondii* was determined by blood samples from pigs of approximately 26 weeks of age slaughtered at three Dutch slaughterhouses [11, 30]. Pigs originated from conventional and organic farms in the Netherlands and Belgium. According to the sampling scheme for determination of antibodies to *T. gondii* [11], a minimum of one and a maximum of six serum samples were taken at slaughter from every delivery of pigs. These samples were tested for the presence of anti-*T. gondii* antibodies using the PrioCHECK™ Toxoplasma Antibody ELISA (Thermo Fisher Scientific Prionics B.V., Lelystad The Netherlands). A cut-off of 20 % positivity (PP) was used to classify serum samples as positive, as recommended by the manufacturer.

Selection of farms to participate in the intervention study occurred dynamically from July 2017 to April 2018. A farm was selected when in this period it had:

- an estimated within-farm seroprevalence of 10 % or higher, which was estimated monthly from the serological data of the previous 12 months using a Bayesian approach in which we accounted for imperfect diagnostic testing [31],
- a minimum of six deliveries in the previous running year,
- the willingness to provide researchers access to the farm and pig stables at least twice.

In this study we used a cross-over trial, with each farm acting as its own control. The cross-over date was the start of the implementation of the intervention measure. The control period was the one year prior to the cross-over date and the intervention period the one year after the cross-over date to control for potential seasonal effects.

### Interventions

After selection, farms received an initial farm visit. During this visit, the control situation was assessed by a researcher, which included a visual inspection of the outside and inside of the pig stables. For this assessment, a questionnaire was set up. This questionnaire was based on the Hazard Analysis Critical Control Points (HACCP) framework of Kijlstra et al. [14], used to identify the most important control measures to prevent, reduce or control the introduction and spread of *T. gondii* on organic pig farms. The questionnaire gathered information about the status of the following topics related to risk factors for *T. gondii* infections in pigs: general farm biosecurity, supply of pigs, outdoor access, presence of cats, feed and water supply and pest control and prevention. On each topic the farm was scored, with a higher score indicating a lower risk for introduction and spreading of *T. gondii* infections.

At the end of the initial visit, the intervention strategy to be implemented on the farm was selected together with the farmer. This intervention strategy differed between farms, each farmer implemented a specific combination of intervention measures best fitting to their farm situation. For each farm, one principal intervention measure was selected at the end of the initial farm visit. The principal intervention measures implemented in this study were:

- Cat neutering: all cats present at the farm were spayed or neutered by a local veterinarian in cooperation with a NGO to prevent the birth of kittens. During their lifetime cats get usually infected once with *T. gondii* and, during this infection, they shed *T. gondii* oocysts in the environment through their feces for only a relative short period of time.
- Professional rodent control: professional rodent control was implemented to reduce the number of potentially infected rodents present on the farm that could spread *T. gondii* oocysts.
- Feed coverage: pig feed was structurally covered to prevent access of cats and rodents to reduce the presence of *T. gondii* oocysts in feed.

The cross-over date of a selected farm was the date on which the principal intervention measure was actually implemented on that farm. For example, Farm 1 was visited for the first time on 14 July 2017. Neutering of the cats present at the farm was selected as principal intervention measure for this farm. Neutering took place on 25 August 2017, therefore this date was the cross-over date for Farm 1. The total study period for this farm ran from 25 to 2016 to 25 August 2018, with 25 August 2016 to 25 August 2017 as control period and 25 August 2017 to 25 August 2018 as the intervention period.

Selected farms were contacted every quarter during the intervention period to assess the progress on the implementation and the expenses of implemented intervention measures. In 60 % of the cases this assessment was done during a farm visit. The remaining assessments were performed through telephone interviews with the farmer. Progress was assessed using the same HACCP-based questionnaire as used in the initial visit. This provided the opportunity to follow the combination of intervention measures (by improvement on points scored for the different topics) implemented at each farm during the intervention period.

A standardized cost statement template was used to register the farmer’s initial one-off expenses (€) and one-off number of hours (labor) needed for implementation of each intervention measure, as well as the monthly expenses (€/month) and monthly amount of labor (hours/month) for running this intervention measure. Own labor costs were valuated at €28.31 per hour [30]. Depreciation, maintenance and interest rates from Blanken et al. [32] were used to estimate the annual costs related to the initial expenses. Depreciation was set at 20.0 % for neutering of cats (assuming neutering is needed every 5 years), 10.0 % for rattraps, boots and boot cleaners, and new doors, 5.0 % for the whey silo, and 2.5 % for the trench silo floor. Maintenance costs were set at 1.3 % for all intervention measures under study, except 0.0 % was used for cat neutering. Annual interest rate was set at 3.5 %.

### Statistical analysis

The odds ratios (OR) of principal intervention measures were estimated using binomial regression in a Bayesian framework as the exponent of the regression parameter (i.e., Exp[β]). The number of positive samples in a delivery *p*_*i*_ were considered ‘successes’ and the number of samples examined as binomial total. The success-rate *p*_*i*_ for principal intervention measure *i* was modelled as

1 $$ logit\left({p}_i\right)={\upbeta}_{0,i}+{\upbeta}_{1,i}\times {Int}_i $$

where β_0_ is the intercept, β_1,i_ the parameter indicating the effect of principal intervention measure *i*, and *Int*_*i*_ an assertion-factor to indicate to what extent the principal intervention measure *i* could have asserted an effect on the seroresponse at slaughter. When principal intervention measure *i* had not yet started, then *Int*_*i*_=0. If principal intervention measure *i* had started, then *Int*_*i*_ was calculated as the number of finishing days between start date of the principal intervention measure *i* and the slaughter date, divided by the baseline length of finishing of 111 days [2]. The proportion was maxed at 1. This approach was chosen to account for the delay in the ability to measure the effect of the intervention at slaughter. An intervention starting the day before slaughter (*Int*_*i*_=1/111) had a lower probability of asserting an effect on the antibody levels at slaughter compared to one having started before fattening (*Int*_*i*_=1).

Model (1) was analyzed separately for each of the three principal intervention measures, neutering of cats (three farms), professional rodent control (one farm), and feed coverage (one farm). Analyses were done in R, using the package rJAGS (JAGS version 4.3.0). All priors were specified to be vague (normal distributions with µ = 0 and precision = 0.0001) in an attempt to have the posterior distribution be influenced mostly by the collected data. Five Markov Chains were run simultaneously for 100,000 iterations with randomly chosen initial values, the first 1,000 iterations were discarded for burn in and the chain stability was monitored by examining the trace plots [33].

## Supplementary Information

**Additional file 1: Figure S1.** Pictures of farm situation on Farm 1. **Figure S2.** Pictures of rodent surveillance cameras on Farm 4. **Figure S3.** Pictures of farm situation on Farm 5.

**Additional file 2: Table S1.** HACCP-based questionnaire and Pig Pointer.

## Data Availability

All data generated or analysed during this study are included in this published article [and its supplementary information files].

## References

[1] Dubey JP (2009). Toxoplasmosis in Pigs—The Last 20 Years. Vet Parasitol.

[2] Foroutan M, Fakhri Y, Riahi SM, Ebrahimpour S, Namroodi S, Taghipour A, Spotin A, Gamble HR, Rostami A (2019). The Global Seroprevalence of Toxoplasma Gondii in Pigs: A Systematic Review and Meta-Analysis. Vet Parasitol.

[3] McAuley JB (2014). Congenital Toxoplasmosis. J Pediatr Infect Dis Soc.

[4] Torgerson PR, Mastroiacovo P (2013). The global burden of congenital toxoplasmosis: a systematic review. Bull World Health Organ.

[5] World Health Organization WHO. Estimates of the Global Burden of Foodborne Diseases 2015.

[6] Bouwknegt M, Devleesschauwer B, Graham H, Robertson L, Giessen J, Lassen B Prioritisation of Food-Borne Parasites in Europe, 2016. Eurosurveillance 2018;23. 10.2807/1560-7917.ES.2018.23.9.17-00161. 10.2807/1560-7917.ES.2018.23.9.17-00161 PMC5840924 29510783

[7] Havelaar AH, van Rosse F, Bucura C, Toetenel MA, Haagsma JA, Kurowicka D, Heesterbeek J, Hans AP, Speybroeck N, Langelaar MFM, van der Giessen JWB (2010). Prioritizing Emerging Zoonoses in The Netherlands. PLoS ONE.

[8] Suijkerbuijk AW, Over EA, Opsteegh M, Deng H, Gils PF, Bonačić Marinović AA, Lambooij M, Polder JJ, Feenstra TL, Giessen JW (2019). A social cost-benefit analysis of two one health interventions to prevent toxoplasmosis. PLOS ONE.

[9] EFSA Technical Specifications on Harmonised Epidemiological Indicators for Public Health. Hazards to Be Covered by Meat Inspection of Swine. EFSA J. 2011:1-125.

[10] Olsen A, Sandberg M, Houe H, Nielsen HV, Denwood M, Jensen TB, Alban L (2020). Seroprevalence of Toxoplasma Gondii Infection in Sows and Finishers from Conventional and Organic Herds in Denmark: Implications for Potential Future Serological Surveillance. Prev Vet Med.

[11] Swanenburg M, Gonzales JL, Bouwknegt M, Boender GJ, Oorburg D, Heres L, Wisselink HJ. Large-Scale Serological Screening of Slaughter Pigs for Toxoplasma Gondii Infections in The Netherlands during Five Years (2012–2016): Trends in Seroprevalence over Years, Seasons, Regions and Farming Systems. Vet Parasitol X 2019;2. 10.1016/j.vpoa.2019.100017. 10.1016/j.vpoa.2019.100017 PMC7458374 32904761

[12] García-Bocanegra I, Simon-Grifé M, Dubey JP, Casal J, Martín GE, Cabezón O, Perea A, Almería S (2010). Seroprevalence and Risk Factors Associated with Toxoplasma Gondii in Domestic Pigs from Spain. Parasitol Int.

[13] Hill DE, Haley C, Wagner B, Gamble HR, Dubey JP (2010). Seroprevalence of and Risk Factors for Toxoplasma Gondii in the US Swine Herd Using Sera Collected During the National Animal Health Monitoring Survey (Swine 2006). Zoonoses Public Health.

[14] Kijlstra A, Meerburg BG, Mul MF (2004). Animal-Friendly Production Systems May Cause Re-Emergence of Toxoplasma Gondii. NJAS - Wagening J Life Sci.

[15] Limon G, Beauvais W, Dadios N, Villena I, Cockle C, Blaga R, Guitian J (2017). Cross-Sectional Study of Toxoplasma Gondii Infection in Pig Farms in England. Foodborne Pathog Dis.

[16] Veronesi F, Ranucci D, Branciari R, Miraglia D, Mammoli R, Fioretti DP (2011). Seroprevalence and Risk Factors for Toxoplasma Gondii Infection on Finishing Swine Reared in the Umbria Region, Central Italy. Zoonoses Public Health.

[17] Villari S, Vesco G, Petersen E, Crispo A, Buffolano W (2009). Risk Factors for Toxoplasmosis in Pigs Bred in Sicily, Southern Italy. Vet Parasitol.

[18] Tenter AM, Heckeroth AR, Weiss LM (2000). Toxoplasma Gondii: From Animals to Humans. Int J Parasitol.

[19] Dubey JP, Weigel RM, Siegel AM, Thulliez P, Kitron UD, Mitchell MA, Mannelli A, Mateus-Pinilla NE, Shen SK, Kwok OCH (1995). Sources and Reservoirs of Toxoplasma Gondii Infection on 47 Swine Farms in Illinois. J Parasitol.

[20] Mateus-Pinilla NE, Dubey JP, Choromanski L, Weigel RM (1999). A Field Trial of the Effectiveness of a Feline Toxoplasma Gondii Vaccine in Reducing T. Gondii Exposure for Swine. J Parasitol.

[21] Dubey JP Toxoplasmosis of Animals and Humans; 2nd ed.; CRC Press: Boca Raton, 2010; ISBN 978-1-4200-9236-3.

[22] Dubey JP, Carpenter JL (1993). Neonatal Toxoplasmosis in Littermate Cats. J Am Vet Med Assoc.

[23] Krijger IM, Ahmed AAA, Goris MGA, Cornelissen JBWJ, Groot Koerkamp PWG, Meerburg BG (2020). Wild Rodents and Insectivores as Carriers of Pathogenic Leptospira and Toxoplasma Gondii in The Netherlands. Vet Med Sci.

[24] Kijlstra A, Meerburg B, Cornelissen J, De Craeye S, Vereijken P, Jongert E (2008). The Role of Rodents and Shrews in the Transmission of Toxoplasma Gondii to Pigs. Vet Parasitol.

[25] Krijger IM, Belmain SR, Singleton GR, Groot Koerkamp PW, Meerburg BG (2017). The Need to Implement the Landscape of Fear within Rodent Pest Management Strategies: Landscape of Fear as Rodent Management Strategy. Pest Manag Sci.

[26] Dubey JP, Murata FHA, Cerqueira-Cézar CK, Kwok OCH, Yang YR. Public Health Significance of Toxoplasma Gondii Infections in Cattle: 2009–2020. J Parasitol 2020;106, 10.1645/20-82. 10.1645/20-82 33326588

[27] van Asseldonk M, van Wagenberg CPA, Wisselink HJ (2017). Break-Even Analysis of Costs for Controlling Toxoplasma Gondii Infections in Slaughter Pigs via a Serological Surveillance Program in the Netherlands. Prev Vet Med.

[28] Garforh C (2015). Livestock Keepers’Reasons for Doing and Not Doing Things Which Governments, Vets and Scientiscts Would Like Them to Do. Zoonoses Public Health.

[29] Van Wagenberg CPA, Van Asseldonk MAPM, Bouwknegt M, Wisselink HJW. Behavioural factors of Dutch pig producers related to control of *Toxoplasma gondii* infections in pigs. Prev Vet Med. 2020. 10.1016/j.prevetmed.2020.104899. 104899]. 10.1016/j.prevetmed.2020.104899 31982804

[30] Hiller A, Oorburg D, Wisselink H, Solt-Smits C, Urlings B, Klein G, Althoff G, Heres L (2013). Prevalence of Mycobacterium Avium in Slaughter Pigs Based on Serological Monitoring Results and Bacteriological Validation. Int J Environ Res Public Health.

[31] Branscum AJ, Gardner IA, Johnson WO (2004). Bayesian Modeling of Animal- and Herd-Level Prevalences. Prev Vet Med.

[32] Blanken K, de Buisonje A, Evers A, Ouweltjes W, Verkaik J, Vermeij I, Wemmenhove H Kwantitatieve Informatie Veehouderij 2020–2021. Handboek 43; Wageningen: Wageningen Livestock Research; 2020.

[33] Gilks WR, Richardson S, Spiegelhalter DJ (1996). Markov Chain Monte Carlo in Practice.

