# Supplementary material for: Effectiveness and costs of interventions to reduce the within-farm Toxoplasma gondii seroprevalence on pig farms in the Netherlands
Source: Porcine Health Manag. 2021 Jul 26;7:44. doi: 10.1186/s40813-021-00223-0 (PMC8311922; doi:10.1186/s40813-021-00223-0)
Supplement: Supplementary file 1 — Additional file 1: Figure S1. Pictures of farm situation on Farm 1. Figure S2. Pictures of rodent surveillance cameras on Farm 4. Figure S3. Pictures of farm situation on Farm 5. [file 40813_2021_223_MOESM1_ESM.docx]

**Supplementary material: Figure S1, S2 and S3**

**
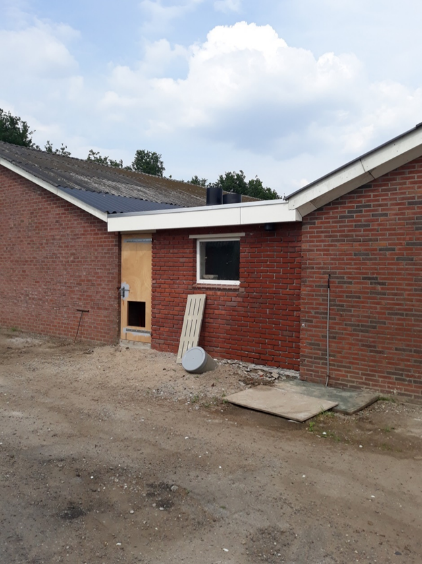

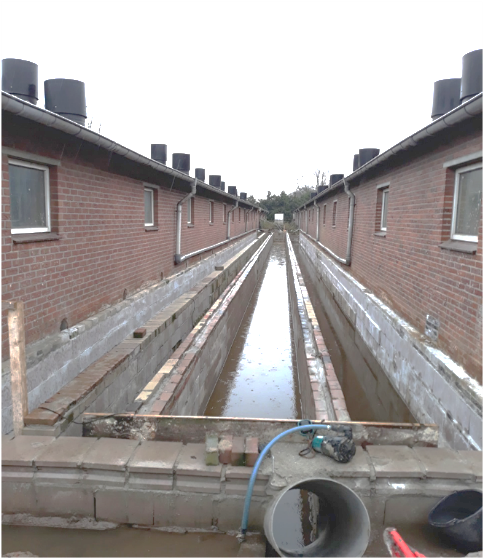

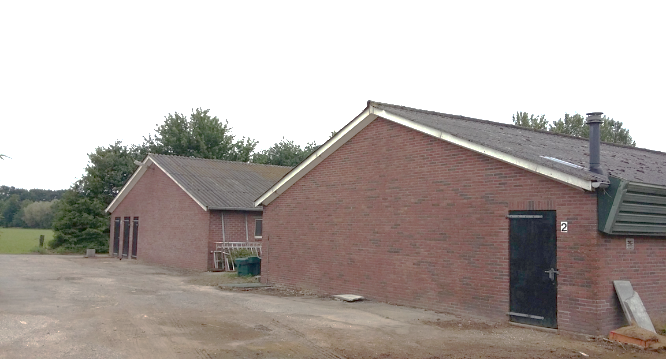

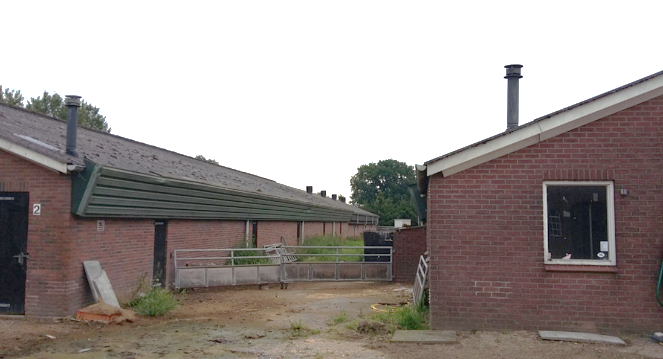
**

**
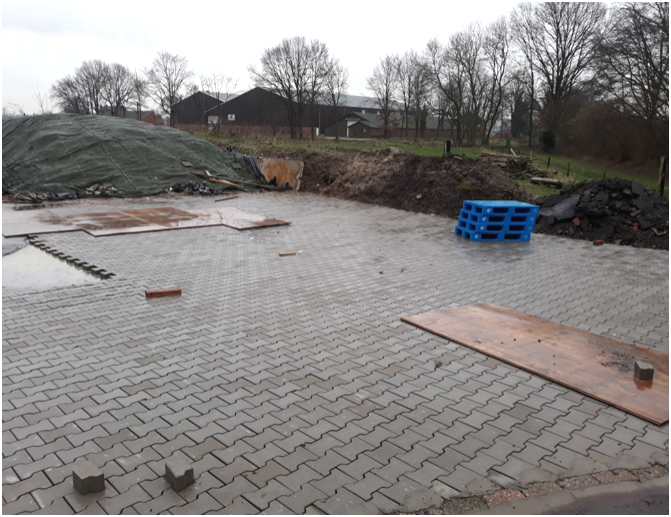

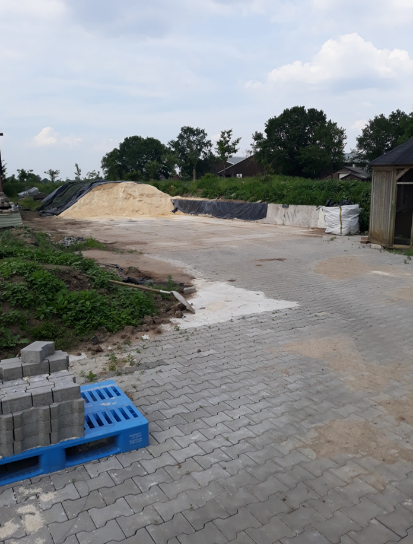

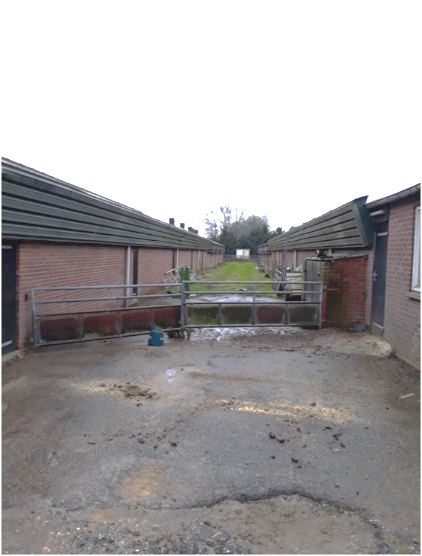
**

**Figure S1:** Pictures of farm situation at Farm 1. Upper two pictures show the situation outside the three stables at the initial visit. The three pictures in the middle show the situation at later stages: a connection was built between stable one and stable two and the farmer tidied up between stable two and three. The two pictures below show the paving of the trench silo.

**
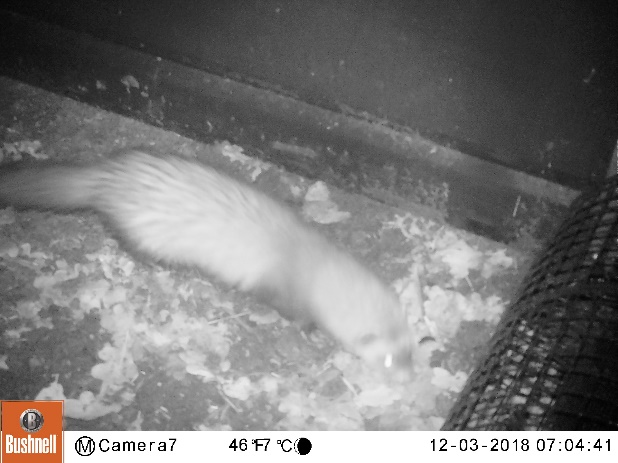

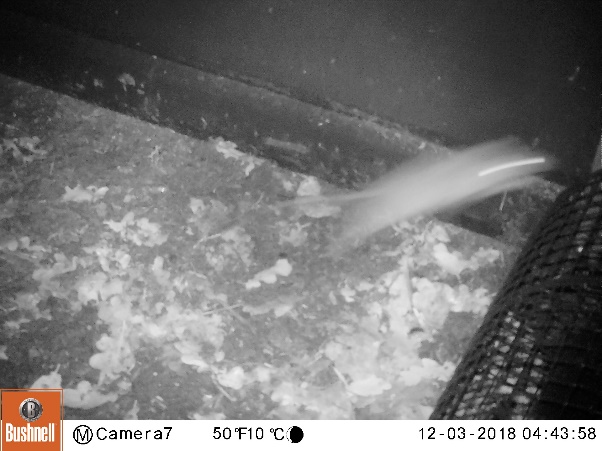

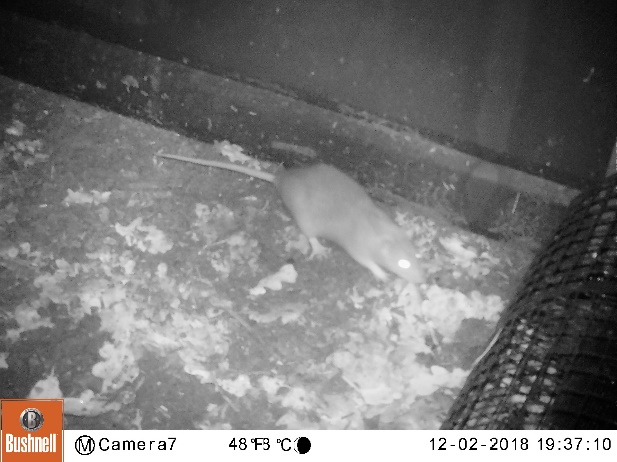
**
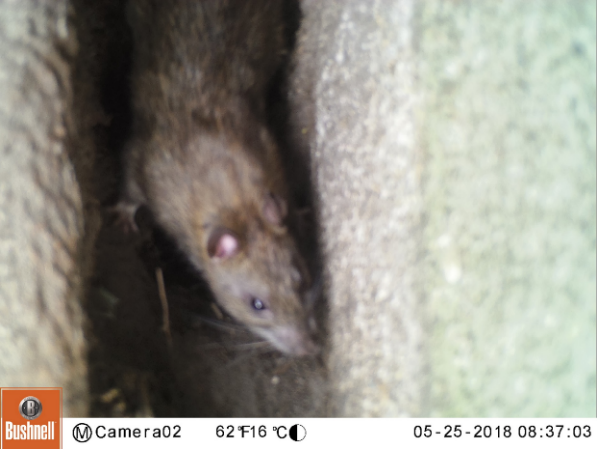

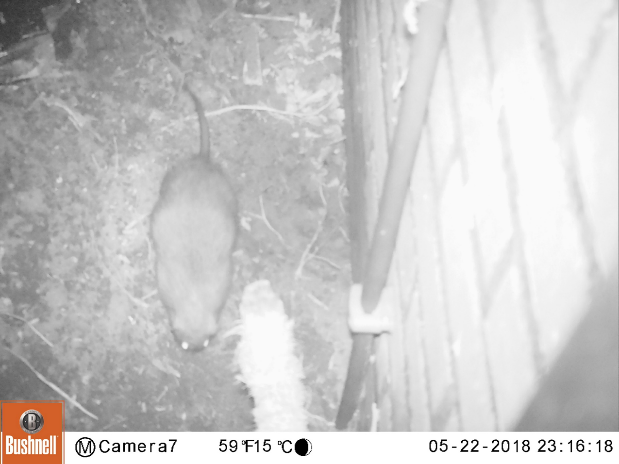


**
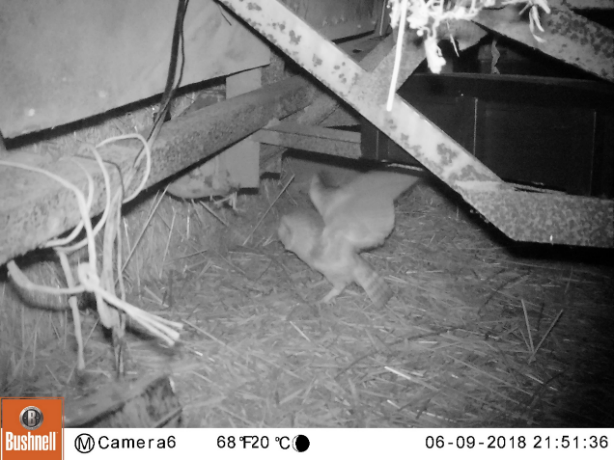
**

**Figure S2:** Pictures taken by rodent surveillance cameras placed at Farm 4 during the two periods of one week camera surveillance. Upper two pictures show the presence of rats on the farm. The two pictures in the middle show the placement of a catch cage (visible in the bottom right of the picture), and how the rat jumped over the cage. In response the farmer closed this route, so the rats could not jump over the cage anymore, which resulted in the capture of five rats. The two pictures below show the presence of natural predators at the farm (an owl and an European polecat).

**
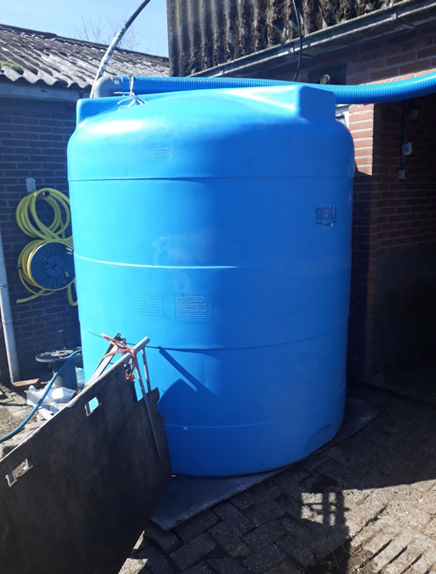

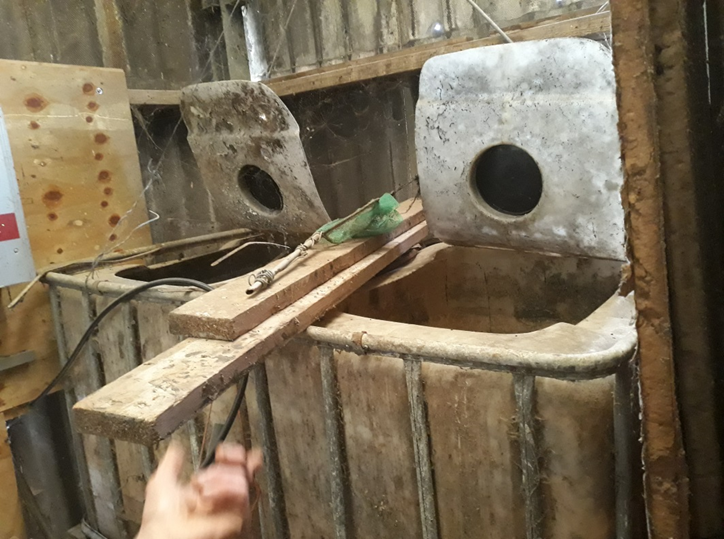
**

**Figure S3:** Pictures of farm situation at Farm 5. The picture on the left shows the old storage of the whey at the initial visit. The picture on the right shows the new storage of whey.
